# Combatting a Tobramycin-resistant *Staphylococcus aureus* outbreak in a neonatal intensive care unit: the impact of hygiene intervention and antibiotic substitution

**DOI:** 10.1186/s12879-026-14481-2

**Published:** 2026-09-18

**Authors:** Anna Jogenfors, Sara Mernelius, Sture Löfgren, Malin Bengnér, Johannes Bengnér, Fredrik Ingemansson, Sofia Wetterbrandt, Andreas Matussek

**Affiliations:** 1 Department of Laboratory Medicine, Region Jönköping County, Jönköping, Sweden; 2 Department of Biomedical and Clinical Sciences, Linköping University, Linköping, Sweden; 3 Department of Infectious Disease Control, Region Jönköping County, Jönköping, Sweden; 4 Department of Medical and Health Sciences, Linköping University, Linköping, Sweden; 5 Department of Pediatrics, Region Jönköping County, Jönköping, Sweden; 6 Department of Microbiology, Division of Laboratory Medicine, Oslo University Hospital, Oslo, Norway; 7 Department of Microbiology, Division of Laboratory Medicine, Institute of Clinical Medicine, University of Oslo, Oslo, Norway

**Keywords:** *Staphylococcus aureus*, Screening, Colonization, Infections, Aminoglycoside resistance, NICU, Antibiotic rotating regimen, Early warning system

## Abstract

**Background:**

Outbreaks of antibiotic-resistant bacteria in neonatal intensive care units (NICUs) is a significant risk to these vulnerable infants. This study describes a decade-long outbreak of tobramycin-resistant *Staphylococcus aureus* (TRSA) and the interventions implemented to try and resolve it.

**Methods:**

A TRSA outbreak was suspected in a Swedish NICU in 2010 when four infants developed bacteremia with TRSA *spa *type t084. The outbreak was confirmed by point prevalence surveys of infants and staff, and monitored through active screening of infants. Several interventions were implemented to combat the outbreak, e.g. hygiene interventions were implemented (2010, 2014), the NICU was reconstructed (2012–2013), tobramycin was substituted for amikacin (2014) and a continued summary of screening results were distributed as a reminder to adhere to hygiene routines.

**Results:**

Retrospective analysis of previous samples revealed TRSA presence as early as 2009. The *S. aureus *prevalence significantly decreased from 71% (2010–2011) to 52% (2013) following the hygiene intervention and pre-pasteurization of donor human milk (DHM). However, the proportion of TRSA persisted. The proportion of TRSA started to significantly decrease only after the ward was reconstructed and the antibiotic regimen was modified, ultimately leading to the apparent resolution of the outbreak in 2019, five years after the change in antibiotic regimen. No TRSA septicemia cases occurred after the change in antibiotic regimen and TRSA in the environmental screenings were no longer detected after 2012.

**Conclusions:**

Our findings underscore the interplay between the ward environment and neonatal colonization, emphasizing that successful outbreak control requires a combination of adherence to hygiene routines, pasteurization of DHM, and a correct antibiotic regimen.

**Clinical trial number:**

Not applicable.

## Background

Yearly, three million newborns suffer from bloodstream infections [1]. Preterm infants without a fully developed immune system are more susceptible to invasive *Staphylococcus aureus* infections than full-term infants [2]. *S. aureus* is a human commensal, and 20 % to 30 % of newborns are colonized [3]. The most important colonization site is suggested to be the anterior nares [4]. However, *S. aureus* is a leading pathogen in neonatal intensive care units (NICUs), causing diseases that ranges from mild skin infections to life-threatening infections [5]. The mortality rate for *S. aureus* infections in NICUs is 1 % [6]. It should be noted that, in neonates methicillin sensitive *S. aureus* (MSSA) infections are reported approximately three times more often than methicillin resistant *S. aureus* (MRSA) [7]. The main risk factors for infections in NICUs are prematurity [2], low birth weight, prolonged hospital stay, and exposure to invasive devices [8, 9]. Akinboyo et al. have shown that invasive respiratory devices, endotracheal tube, nasal cannula/mask increase the risk of recurrent *S. aureus* colonization [10].

*S. aureus* causes outbreaks in NICUs [11] and healthcare-associated infections in infants admitted to NICUs lead to longer hospital stays [6]. Staff members and parents are important transmission reservoirs and cross-transmission between staff members and infants seems to be an important route [12, 13]. In addition, inanimate surfaces may be contaminated with *S. aureus* [14]. The importance of multimodal hygiene interventions to prevent bacterial transmission has been documented [12, 15, 16].

Antibiotic resistance is a global threat and it was estimated that 1.14 million deaths were attributable to bacterial antimicrobial resistance in 2021. However, a significant reduction in attributable direct deaths have been seen in children < 5 years of age [17]. Knowledge of the antibiotic resistance pattern of *S. aureus* in the local ward is crucial, and surveillance programs on antibiotic resistance are important to enable early detection of emerging resistant strains, monitoring of transmission patterns, and evaluation of infection control interventions, all of which are critical in vulnerable populations such as neonates [18]. Antibiotic selection pressure favors the surveillance and spread of resistant strains within healthcare settings [19]. To eliminate resistant strains, the use of rotating antibiotic regimens based on knowledge of the local resistance pattern is a possibility [20–22].

In 2010, four cases of bacteremia caused by a tobramycin-resistant and methicillin susceptible *S. aureus* (TRSA, MSSA) of *spa* type t084 were identified in the NICU at Ryhov County Hospital, Jönköping, Sweden. Screening and molecular typing of strains isolated through point prevalence surveys revealed an outbreak of TRSA of *spa* type t084 among the infants during a period when tobramycin (in combination with penicillin G) was used in the empirical antibiotic regiment, primarily targeting Gram-negative bacteria, but also used for *S. aureus* coverage in Sweden [23].

In this study, we describe a decade-long endemic outbreak of TRSA *spa* type t084 in a Swedish NICU and the measures taken to attempt to eradicate it. Our findings suggest that ward reconstruction and antibiotic substitution was temporally associated with the resolution of the outbreak.

## Material and methods

### Clinical setting

The study was conducted in Jönköping County, with an average population of 350 000 inhabitants during the study period (2010 through 2019). In Jönköping County there is one neonatal intensive care unit (NICU) which serves all three hospitals in the region. During the study period 283 to 411 newborns were admitted yearly to the NICU at Ryhov County Hospital, Jönköping, Sweden. Approximately 5 % are born outside Jönköping. The NICU provides care for premature infants from gestational week 27 + 0, with the option of mechanical ventilation. During the study period, the infants were born at median gestation week 29 + 4 (range 23 + 3 - 42 + 2), with a median length of stay of 43 days.

In 2010, the NICU had 13 beds, of which four beds for intensive care (divided into two double rooms), four beds were for intermediate care (one four-bed room) and five were rooms with family joint care beds. After a previously decided (2007) reconstruction in 2012–2013, the NICU has 16 beds with two more beds for intensive care (one new double room) and one more room with a family joint care bed.

### Sampling of infants

In April (*n* = 1) and May (*n* = 3) of 2010, four cases of *S. aureus* bacteremia (three septicemia and one peripheral venous catheter infection) caused by TRSA (MSSA) of *spa* type t084 were identified in infants in the NICU at Ryhov County Hospital. This led to the collection of point prevalence screening samples from all infants admitted to the NICU on five separate occasions between 2010 and 2011.

An active screening program was initiated in 2012, with the aim of enabling early detection of colonization and characterization of local antibiotic resistance patterns. The program encompassed both Gram-positive and Gram-negative bacteria and was not restricted to MRSA or multidrug-resistant Gram-negative bacteria, differing from a clinical culture and screening for multi-resistant bacteria.

**Fig. 1:**
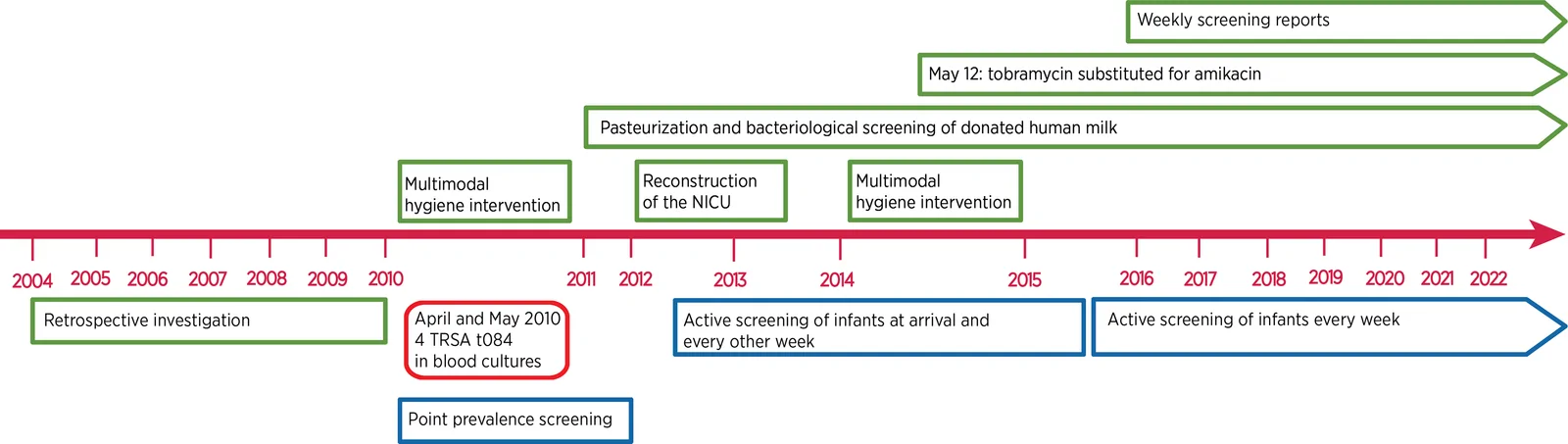
Timeline depicting the outbreak of tobramycin-resistant *S. aureus* (TRSA) *spa* type t084 and the interventions implemented to try and resolve the outbreak

The active screening program included collection of samples from infants every two weeks (on Tuesdays, regardless of infant age) and upon admission from other NICUs. In 2015, the program was intensified through sampling every week (Fig. 1), to make it easier for the staff to adhere to the screening protocol. All results from the active screening program were reported to the clinicians through the laboratory information system.

Samples from infants in the point prevalence screenings and the active screening program were collected from anterior nares, umbilicus, perineum, and any catheters or skin lesions using swabs (Copan Italia, Brescia, Italy). Detection of *S. aureus* in samples from perineum was excluded from the active screening in 2016, due to financial aspects.

### The interventions

To manage the TRSA outbreak, several multimodal interventions were implemented as shown in the timeline (Fig. 1).

The multimodal hygiene interventions, in 2010 and 2014, were done in collaboration between staff of the department of infection prevention and control, the microbiology laboratory and the NICU, with the aim to improve adherence to standard precautions and hygiene guidelines. The interventions included lectures on hospital hygiene and workshops where staff members reflected on statements regarding hygiene guidelines, in their daily work. They also encompassed infection control audits. These included questions on how staff got knowledge about hygiene guidelines, an inspection and discussion on the local possibilities to adhere to these guidelines and feedback to staff and management for further hygiene improvement [15]. Bacterial screening and pasteurization of donor human milk (DHM) were initiated in 2011. On May 12th 2014, tobramycin was substituted for amikacin (based on *S.aureus* susceptibility pattern), in combination with penicillin G (used both before and after the antibiotic substitution) as an empiric antibiotic regimen. The screening program was intensified with screening and reports on bacterial findings and TRSA proportions every week in 2015.

Compliance with barrier precautions (hand disinfection before and after contact with infants, correct use of gloves, plastic apron or other protective equipment) was measured through observations monthly from 2011 and throughout the study period [24].

### Sampling of staff

Point prevalence screening samples from the anterior nares and throat of staff members, using swabs (Copan Italia), were collected once (voluntary and anonymous) in 2010. Neither follow-up cultures nor decolonization protocols were implemented for colonized staff.

### Environmental and air sampling

Staff from the department of infection prevention and control collected environmental samples from clinical equipment and frequently touched surfaces in the ward and staff member areas. Environmental sampling, was done on four separate occasions between 2010 and 2019.

Environmental sampling from surfaces was done with swabs (Copan Italia) or in-house contact blood agar plates (100 g of dehydrated Difco Columbia blood agar base (279240, Thermo Fischer Scientific, Waltham, MA), 2.27 L deionized water and between 135 and 165 mL of human blood). The mixture was dispensed in plates (5 cm in diameter), in a convex form.

Between 2012 and 2013 air sampling was done on 13 occasions (due to the reconstruction of the unit), each time collecting five to nine samples, through collection of 1 000 L of air on a blood agar plate using a surface air sampler (air IDEAL, bioMeriéux AB, Marcy l’Etoile, France).

### Bacterial cultures

Swabs from infants and staff members were inoculated on BBL CHROMagar Staph aureus agar media (BD Diagnostics, Sparks, Md.), with a tobramycin disk in the primary streak (as a screening for tobramycin-resistance, always followed by antibiotic susceptibility testing, for details see below), and incubated aerobically at 37 °C for 20 to 24 hours.

Swabs from environmental sampling were inoculated on blood agar plates. These plates, contact blood agar plates and blood agar plates from air sampling, were incubated aerobically at 37 °C for 48 hours.

DHM were cultured and semi quantified on blood agar plates (1 µl and 100 µl) and on BBL CHROMagar Staph aureus agar media (BD Diagnostics) (100 µl) and ChromID CPS (bioMeriéux AB) (100 µl). Growth of skin flora was accepted in DHM. The following bacteria were not accepted: *Streptococci* (group A, B and C/G), TRSA, MRSA, ESBL producing bacteria, VRE, *Salmonella*, *Listeria monocytogenes* and multiresistant Gram-negative bacteria (resistant to ≥ 3 different antibiotic classes). Fucidic acid-resistant *S. aureus* (FRSA) was not accepted until mid-2014. *Enterobacteriaceae* were accepted below 10^4^ CFU/mL. All other bacteria were accepted if the quantity was below 10^4^ CFU/mL.

*S. aureus* was confirmed by MALDI-ToF MS (Bruker GmbH, Bremen, Germany).

### Antibiotic susceptibility testing

Antibiotic susceptibility testing (AST) was done by disk diffusion on Mueller-Hinton Agar (Bio-Rad, Hercules, CA)), with antibiotic disks (Oxoid) according to the European Committee on Antimicrobial Susceptibility Testing (EUCAST) Disk Diffusion Test Methodology applicable at the time (https://www.eucast.org/bacteria/document-archive/). The following antibiotic disks were used for surveillance: cefoxitin (30 μg), tobramycin (10 μg), amikacin (30 μg), gentamicin (10 μg) and netilmicin (30 μg). Methicillin resistance in isolates resistant to cefoxitin was verified by PCR detection of *mecA* [25].

### *spa* typing

*spa* typing was done as previously described [26]. Sanger sequencing was done by Eurofins Genomics (Ebersberg, Germany) and each isolate was assigned a *spa* type by RidomStaphType software (v1.5.21, Ridom GmbH, Würzburg, Germany). Only isolates of the same *spa* type were considered related [27]. The data for this study have been deposited in the European Nucleotide Archive (ENA) at EMBL-EBI under accession number PRJEB108429 (https://www.ebi.ac.uk/ena/browser/view/PRJEB108429).

### Retrospective investigation

The laboratory information system was reviewed retrospectively for records of the phenotype TRSA in clinical samples from infants in the NICU (isolates not stored) and in environmental samples from previous outbreak investigations (isolates stored) from 2004 through 2009.

### Infections with TRSA in infants

All clinical cultures (collected based on a suspected infection) from infants admitted to the NICU between May 11th 2012 and December 31st 2019, with growth of TRSA, were identified in the laboratory information system. An examination of the medical records of these infants was done. Data from earlier clinical cultures were unavailable due to a change in the laboratory information system.

### Statistical analyses

Excel 2010 (Microsoft, Redmond, WA) was used for statistical analyses. A two-sided chi-square test and Fischer’s exact test with one degree of freedom were used for the evaluation of proportions. The R Software version 4.4.1 (R Foundation for Statistical Computing) was used for logistic regression and the interpretation of the *S. aureus* prevalence and TRSA proportion. A *p* value of ≤ 0.05 was considered significant.

### Ethics

The Swedish Ethical Review Authority (The Regional Ethical Review Board in Linköping, Sweden DNR:2017/187–31) approved the study. The authority waived written informed consent, since infants were sampled within the framework of the routinely performed patient safety work. Written consent to take part of medical journals of patients was obtained from the head of the department of pediatrics. The study adhere to the Declaration of Helsinki.

## Results

### Cultures from infants

#### Retrospective investigation

In clinical non-blood cultures three TRSA of *spa* type t084 were detected from September through November 2009.

#### Outbreak investigation

Figure 1 illustrates the details of the outbreak and the interventions. The prevalence of *S. aureus* and the proportion of TRSA among infants admitted to the NICU during the outbreak are shown in Table 1. The *S. aureus* prevalence decreased from 72 % (2011) to 52 % (2013) (*p* = 0.00003) after the hygiene intervention in 2010 and the start of pre–pasteurization of DHM 2011. After 2013, no further reduction in *S. aureus* prevalence was observed (Table 1).

**Table 1:** Prevalence of *S. aureus* and tobramycin-resistant *S. aureus* (TRSA) in point prevalence and active screening samples from 2010 through 2021 in the NICU

		No. of infants		
			Colonized with	
**Type of sampling**	**Sampling time**	**Sampled**	***S. aureus*** **(%)**	**TRSA (%)**
Point prevalence	2010	52	37 (71)	22 (59)
	2011	18	13 (72)	7 (54)
Active screening	2012 (May-Dec)	129	80 (62)	45 (56)
	2013	164	85 (52)	24 (28)
	2014	348	146 (42)	33 (23)
	2015	254	130 (51)	30 (23)
	2016	313	159 (51)	26 (16)
	2017	338	169 (50)	3 (2)
	2018	298	162 (53)	9 (6)
	2019	291	154 (53)	8 (5)
	2020	308	147 (48)	0 (0)
	2021	294	163 (55)	0 (0)

**Fig. 2:**
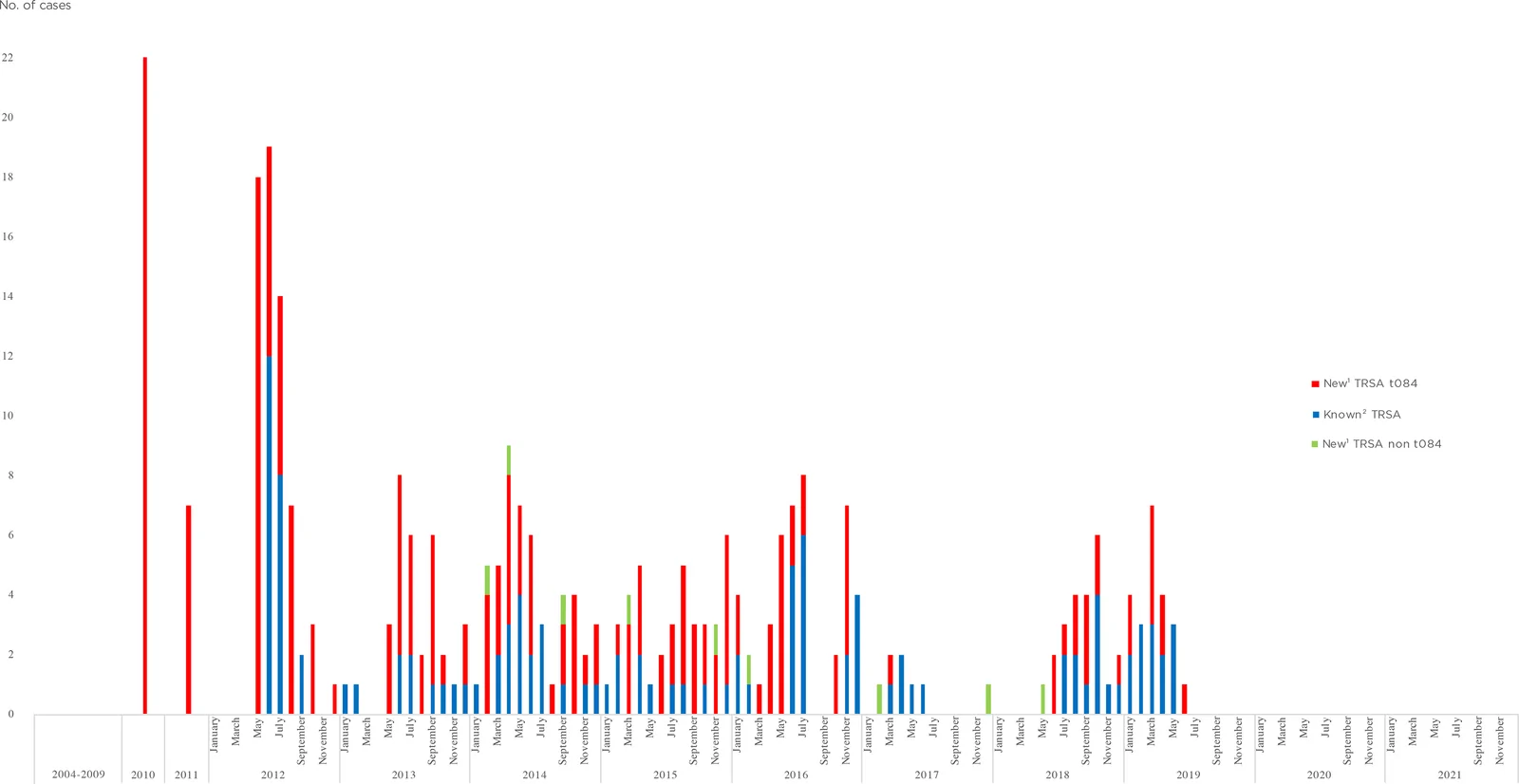
Tobramycin-resistant *S. aureus* (TRSA) cases over time. ^1^First detection of TRSA in a patient not previously known to be colonized. ^2^Patients with previously confirmed TRSA colonization

The TRSA proportion decreased after the reconstruction of the ward (*p* = 0.0009) and after the antibiotic substitution (*p* = 2×10^−16^). No TRSA t084 were detected among the infants in the NICU between July 2017 and June 2018. TRSA t084 reappeared in August 2018 and was detected through June 2019 in 13 infants. Thereafter it was not present to the end of the study period (throughout 2019), neither during a two year follow-up period (Fig. 2).

The average compliance with barrier precautions between 2011 throughout the study period was 93 % (data not shown).

### Cultures from staff

The prevalence of *S. aureus* among staff in 2010 was 37 % (25 out of 68), and two of them were colonized with TRSA t084.

### Cultures from donor human milk

Of the 393 samples analysed during 2012–2019, 95 were discarded (24.2 %). TRSA was detected in three separate years, 2012, 2014, and 2019, with the highest number of findings observed in 2014 (*n* = 3).

### Cultures from the environment and air

#### Retrospective investigation

No TRSA were found in the environmental samples (*n* = 21) collected in 2005 during an MRSA outbreak; however, two tobramycin-susceptible *S. aureus* (TSSA) of *spa* type t084 were found (data not shown). Follow-up environmental sampling in 2006 (*n* = 17) revealed no *S. aureus*. From January 2008 through February 2009, *S. aureus* was found in 21 of 118 samples during seven sampling occasions, none of which were TRSA. In December 2009 *S. aureus* was found in 5 out of 22 samples, of which three were TRSA t084.

#### Outbreak investigation

The results from the environmental samplings from 2010 to 2019 are shown in Table 2. Before the reconstruction, 82 environmental samples were collected, and TRSA was found in 9 of these samples. During the end of the reconstruction, in 2012, four TRSA were found in the 70 air samples collected. After the reconstruction, TRSA was not detected in any of the 57 environmental and 15 air samples collected.

**Table 2:** Prevalence of *S. aureus* and tobramycin-resistant *S. aureus* (TRSA) in environmental and air samples from 2010 through 2019 in the NICU

Type of sampling	Sampling time	No. of samples	*S. aureus ***(%)**	TRSA (%)
Environment	2010	23	2 (9)	1 (50)
Environment	2011	59	14 (24)	8 (57)
Air	2012	70	13 (19)	4 (31)
Air	2013	15	0 (0)	0 (0)
Environment	2014	11	0 (0)	0 (0)
Environment	2019	46	1 (2)	0 (0)

### Antibiotic susceptibility

One environmental *S. aureus* isolate, collected in 2010, was resistant to tobramycin, amikacin, gentamicin and netilmicin (not found in any infant). All other *S. aureus* isolates tested during the study period were susceptible to amikacin, gentamicin and netilmicin, independent of tobramycin resistance. During the study period ten infants were colonized with MRSA, but no transmission was seen.

Tobramycin resistance in Gram-negative organisms were detected in 106 out of 473 cultures between 2012 and 2019 in *Escherichia coli* (71/211), *Proteus mirabilis* (33/59) and *Morganella morganii* (2/3). Amikacin resistance were not detected in any of the 305 cultures growing Gram-negatives tested for amikacin between 2014 and 2019.

### TRSA *spa* types

TRSA was isolated from 29 infants during five point prevalence screening occasions in 2010 and 2011, and all the isolates were *spa* type t084. In the active screening program, TRSA was isolated from 80 infants before the antibiotic substitution, whereof 78 isolates were *spa* type t084 and the remaining two were *spa* types t021 and t7094. After the substitution TRSA was isolated from 94 infants, whereof 87 isolates of *spa* type t084, two each of t346 and t091 and one each of t2530, t19104 and t5196.

TRSA of *spa* type t084 resulted in secondary cases, no TRSA of other *spa* types resulted in secondary cases (Fig. 2).

All TRSA isolated from the environment, air, and staff were of *spa* type t084.

### Infections with TRSA in infants

From May 11th 2012 through December 31st 2019, 2746 infants were admitted to the NICU (Table 3). Before and after the antibiotic substitution on May 12th 2014, the proportion of patients with suspected infections was the same. However, the proportion of TRSA in clinical samples gradually decreased. No infants presented with TRSA septicemia after antibiotic substitution (Table 3). The overall incidence of healthcare-associated infections (HAIs) in the NICU defined as infections with onset more than two days after admission were observed in a lower proportion after (1 to 3 %) than before (2 to 7 %) the antibiotic substitution (Table 4).

**Table 3:** Tobramycin-resistant *S. aureus* (TRSA) infections among infants in the neonatal intensive care unit (NICU) before and after antibiotic substitution

Number of infants	2012–05-11 to 2014–05-11	2014–05-12 to 2019–12-31	*p*
admitted to the NICU	611	2135	
with a sample based on clinical indication	119	401	0.75*
with TRSA in clinical samples	11	13	0.006**
with a TRSA septicemia	4	0	0.02***

*comparing infants with a clinical sample to admitted infants

**comparing infants with TRSA in clinical samples to infants with a clinical sample

***comparing infants with a TRSA septicemia to infants with TRSA in clinical samples

**Table 4:** Inpatient data for infants admitted between 2010 and 2019

Year	2010	2011	2012	2013	2014	2015	2016	2017	2018	2019
Number of infants in care	283	299	246	294	339	319	356	411	354	347
Number of infants receiving tobramycin			99	114	41					
Days of therapy (DOT) with tobramycin			492	560	193					
Tobramycin DOT per infant receiving tobramycin			4.97	4.91	4.71					
Number of prescriptions with tobramycin			107	128	42					
Number of infants receiving amikacin					85	110	132	163	135	149
Days of therapy (DOT) with amikacin					429	468	580	764	513	534
Amikacin DOT per infant receiving amikacin					5.05	4.25	4.39	4.69	3.80	3.58
Number of prescriptions with amikacin					100	119	148	189	171	188
Healthcare-associated infections^*^	21	13	12	5	11	6	7	10	11	2

*Healthcare-associated infections are defined as infections with onset more than two days after admission

## Discussion

A TRSA outbreak was suspected when four infants at the NICU tested positive for TRSA in their blood cultures within a two months period. This suspicion was confirmed by identifying that all isolates were of *spa* type t084. The extent of the outbreak was further assessed through screening of infants, staff, and environmental and air samples, revealing that up to 59 % of the infants colonized with *S. aureus* carried TRSA. Screening of infants and staff has been frequently used as measures to terminate NICU outbreaks [6]. A recent publication from the Centers for Disease Control and Prevention (CDC) recommends active surveillance for *S. aureus* colonization in NICU patients in an outbreak setting [28]. Outbreaks with resistant *S. aureus* in NICUs may be fatal and prolong the average length of hospital stay [29]. Narui et al. [30] demonstrate that the hospital environment acts as a dynamic reservoir, that is rapidly colonized by the staff’s own bacterial flora through human movement and shared infrastructure. 

The *S. aureus* prevalence was 72 % initially and decreased to 52 % after the interventions comprising a hygiene intervention in 2010 and pre-pasteurization of DHM in 2011. Notably, the observed decrease in *S. aureus* prevalence contrasted with the high compliance rates, suggesting that hygiene routine assessments were insufficient. However, approximately 50 % of the infants were still colonized with *S. aureus* until the end of the study period. This may reflect the sampling from multiple sites, which has previously revealed high colonization rates [8]. This rate is comparable with the previously described *S. aureus* colonization rate in newborn full-term infants and among the parents, from the same county [12]. *S. aureus* colonization is a risk factor for *S. aureus* infection [4]. The weekly screening reports were posted for all staff members to see and may have worked as a nudge; a small but constant reminder of the continuous transmission of TRSA [31]. Active surveillance is one of the most effective tools for reducing nosocomial infections in ICUs [32], consistent with findings from a previous study conducted in a NICU in Spain [33]. 

The reconstruction of the ward was associated with a statistically significant reduction in TRSA. In a previous study from Taiwan, the transition to a new NICU directly contributed to a significant reduction in hospital-acquired infections [34]. Aminoglycoside-modifying enzymes commonly mediate aminoglycoside resistance in *S. aureus*. The specificity of these enzymes varies where some confer resistance to specific aminoglycosides (e.g., tobramycin but not amikacin) [35], which is consistent with the observed susceptibility to amikacin in our study. Resistance may be acquired through chromosomal mutations, but horizontal gene transfer (e.g. transposons or plasmids) more frequently mediates clinically important resistance [35]. However, since no genotypic characterization was done in this study, the specific mechanism of tobramycin resistance in our outbreak strain remains unknown.

As no concomitant aminoglycoside resistance was detected in TRSA isolates, a change in the empiric aminoglycoside antibiotic regimen was possible, and implemented 12th May 2014. Following the antibiotic substitution, phenotypic tobramycin resistance in *S. aureus* progressively declined and was eventually no longer detected by routine surveillance. However, phenotypic reversion cannot be excluded, and eradication of the resistant strain cannot be confirmed without genotypic characterization.

It has been demonstrated that antibiotic selection pressure has an impact on the pattern of antibiotic resistance [36, 37]. The proportion of TRSA in clinical samples decreased, and no cases of septicemia with TRSA were detected after the antibiotic substitution and the proportion of HAIs also decreased (Table 4). An antibiotic rotation regimen should be considered during endemic outbreaks caused by resistant strains in NICUs [38]. Knowledge of the antibiotic resistance pattern is crucial in choosing an empirical antibiotic regimen [36]. Antibiotic resistance surveillance systems are important on a global, national, and local basis, and an early warning system might facilitate the selection of antibiotic regimens [39].

Previous studies on MRSA outbreaks in NICUs have shown that colonized staff could be a source of transmission to infants [40, 41]. Decolonization of staff has proven efficient in stopping MRSA outbreaks [42]. It might be that the two staff members who carried TRSA t084 played a role in the TRSA transmission, however this was not studied because samples were collected anonymously. Despite the recognized role of healthcare workers in transmission, national guidelines and established practice did not advocate repeated screening of all staff. Accordingly, the acceptability of such a measure was deemed low. Retrospective investigation of clinical samples revealed three TRSA isolates of *spa* type t084 in late 2009, prior to the detection of the outbreak. Three TRSA t084 were also isolated from environmental samples collected in late 2009 in the NICU during an outbreak of extended-spectrum beta-lactamase (ESBL) producing *Citrobacter freundii*. This indicates that environmental screening could be used to identify circulating resistant strains [43]. These potential index isolates of the outbreak were not noticed until the retrospective investigation in 2010. The outbreak strain was thus introduced at the latest in 2009. The antibiotic selection pressure of tobramycin could have facilitated its spread. Tobramycin-susceptible *S. aureus* TSSA of the same *spa* type was prevalent in previous studies during the same time period from the same geographical area [24, 44, 45]. It is possible that this TSSA acquired tobramycin resistance; however, this was not examined in the current study.

*S. aureus* of *spa* type t084 caused secondary cases on several occasions, unlike the other *spa* types detected during the outbreak. The actual route of transmission was not investigated in this study, and there are few reports on this issue [46]. However, the most well documented transmission route is via the hands of healthcare workers

[47]. A previous study indicated that 60 % of *S. aureus*-colonized full-term infants were colonized with the same strain as their parents [24]. The contribution of parental colonization to the outbreak cannot be excluded due to the absence of parental screening.

A key limitation is the inherent complexity of conducting a retrospective study within routine clinical practice were outbreak management are constrained by clinical limitations and standard-of-care considerations. Specifically in this study, the alternation between air sampling and environmental culturing, which are not directly comparable limits the interpretation of environmental surveillance results. Also, the simultaneous implementation of multiple interventions precluded an evaluation of the efficacy of individual measures. The statistical analyses rely on comparisons of proportions before and after interventions, which provide limited evidence for temporal effects. Other study limitations include the exclusion of TSSA infections from the analysis and that Staff screening were only performed once, which is another limitation. Unfortunately patient-level clinical data associated with TRSA colonization required for multivariate logistic regression analysis were not available, as collection of these data was not covered by the ethical approval for the study. The study does not provide any quantitative information regarding antibiotic use, which is a limitation. At the beginning of the outbreak *spa* typing were gold standard for *S. aureus* typing and whole genome sequencing (WGS) were introduced later. WGS would have provide a better discriminatory power and a possibility for further genotypic characterization. Nevertheless, the interventions successfully reduced the *S. aureus* prevalence, and the TRSA phenotype was eventually no longer detected, apparently resolving the TRSA outbreak.

## Conclusions

In summary, a combination of different interventions was required for the apparent resolution of a decade-long TRSA outbreak that initially was undetected for at least several months. The hygiene intervention introduced in 2010, together with pasteurization of DHM, was associated with a reduction in *S. aureus* prevalence in infants. Reconstruction of the ward and antibiotic substitution, finally led to no more detection of the TRSA phenotype, apparently resolving the TRSA outbreak. Continued hygiene efforts are essential for gaining and maintaining a low transmission risk.

## Data Availability

The data for this study have been deposited in the European Nucleotide Archive (ENA) at EMBL-EBI under accession number PRJEB108429 (https://www.ebi.ac.uk/ena/browser/view/PRJEB108429).
